# A Rare Case of Endomyocardial Fibrosis in an Adult

**DOI:** 10.7759/cureus.117726

**Published:** 2026-10-10

**Authors:** God-power Nobime, Yasmine Nnomo Zanga, Nadia Fellat

**Affiliations:** 1 Cardiology, Faculty of Medicine and Pharmacy of Rabat, Mohammed V University of Rabat, Ibn Sina University Hospital Center, Rabat, MAR

**Keywords:** cardiac magnetic resonance, echocardiography, endomyocardial fibrosis, heart failure, restrictive cardiomyopathy

## Abstract

Endomyocardial fibrosis (EMF) is a form of restrictive cardiomyopathy characterized by the presence of fibrotic tissue in the endomyocardium of the ventricles. Even though rare, a high prevalence of this disease is found in the developing world, notably sub-Saharan Africa, Asia, and some parts of South America. Although the origin is not well known, poverty, diet, and infection are known to be implicated in the pathophysiology of the disease.

Transthoracic echocardiography (TTE) has been the first-line modality for assessing the disease over the years before the advent of CMR, which has shown higher accuracy than TTE in the diagnosis of EMF. We present a case study of EMF in a 43-year-old man, a Maghreb trader, diagnosed by cardiac magnetic resonance imaging.

## Introduction

Endomyocardial fibrosis (EMF) is a restrictive cardiomyopathy characterized by fibrotic involvement of the ventricular endocardium, typically affecting the ventricular apices. Progressive endocardial fibrosis and apical obliteration can impair ventricular filling and lead to heart failure, atrioventricular valve regurgitation, atrial enlargement, and intracardiac thrombus formation. It has classically been described in tropical regions, particularly in sub-Saharan Africa, Latin America, and parts of Asia [1,2]. Nevertheless, sporadic cases have also been reported outside the main endemic regions, including North Africa [3,4]. Its etiology remains incompletely understood and likely involves a combination of environmental, nutritional, immunological, infectious, and possibly genetic factors [5,6]. Transthoracic echocardiography (TTE) plays a central role in the initial evaluation of EMF and may demonstrate apical obliteration, endocardial fibrosis, atrioventricular valve regurgitation, atrial enlargement, and intracardiac thrombi [7,8]. Cardiac magnetic resonance imaging (CMR) provides complementary information through tissue characterization, particularly the distribution of endocardial fibrosis and late gadolinium enhancement, and helps differentiate enhancing fibrotic endomyocardium from non-enhancing thrombus [9].

We report an unusual case of EMF in a 43-year-old Maghrebi man with no recent travel to a tropical endemic region, presenting with severe left ventricular systolic dysfunction and multiple apical thrombi. This case highlights the diagnostic value of multimodality imaging and the diagnostic challenges of EMF occurring outside its traditional epidemiological setting.

## Case presentation

A 43-year-old Maghrebi man, working as a trader, was admitted in October 2022 for progressive dyspnea. He lived in an urban area and denied recent travel to tropical regions. His family history was notable for dilated cardiomyopathy in an older sister, diagnosed by echocardiography without genetic testing. Cardiovascular disease was also reported in his consanguineous paternal grandparents; both were deceased, and no medical records were available.

On admission, physical examination showed signs of congestive heart failure. Initial laboratory testing showed elevated NT-proBNP (2,863 ng/L), troponin (3.466 ng/mL), and C-reactive protein (58 mg/L). The complete blood count was unremarkable, with an eosinophil count of 0.2 G/L (3%), confirming the absence of peripheral eosinophilia (Table 1). 

**Table 1:** Admission laboratory findings MCV: mean corpuscular volume; MCH: mean corpuscular hemoglobin; NT-proBNP: N-terminal pro-brain natriuretic peptide; HF: heart failure

Parameter	Result	Unit	Reference range
Hemoglobin	14.2	g/dL	12-17 g/dL
Hematocrit	42	%	36-50%
Red blood cells	4.7	T/L	4.0-5.7 T/L
MCV	89	fL	80-100 fL
MCH	30	pg	27-32 pg
MCH concentration	34	g/dL	32-36 g/dL
White blood cells	6.8	G/L	4-10 G/L
Neutrophils	4.0 (59%)	G/L	1.5-7.5 G/L
Lymphocytes	2.0 (29%)	G/L	1.0-4.0 G/L
Monocytes	0.5 (7%)	G/L	0.2-1.0 G/L
Eosinophils	0.2 (3%)	G/L	<0.5 G/L
Basophils	0.1 (1%)	G/L	<0.1 G/L
Platelets	250	G/L	150-400 G/L
C-reactive protein	58	mg/L	<5 mg/L
High-sensitivity troponin	3.466	ng/mL	<0.034 ng/mL (male)
NT-proBNP	2,863	ng/L	<125 ng/L (chronic HF); <300 ng/L (acute HF)

Electrocardiography showed sinus tachycardia, a normal QRS axis, left-sided chamber enlargement, and pseudo-Q waves in V1-V2. The original ECG tracing from the 2022 admission is no longer available.

On Day 3, transthoracic echocardiography demonstrated severe global left ventricular systolic dysfunction with a left ventricular ejection fraction of 27% and three thrombi attached to the left ventricular apex (Figure 1). The left atrium was dilated, whereas the right atrium was normal in size. Right ventricular systolic function was preserved. The E/e′ ratio was 10, tricuspid regurgitation was physiological, and no significant valvular disease was identified. The inferior vena cava was non-dilated with preserved respiratory variation. Anticoagulation with a vitamin K antagonist was initiated. On Day 5, coronary angiography showed no significant obstructive coronary artery disease (Figure 2).

**Figure 1:**
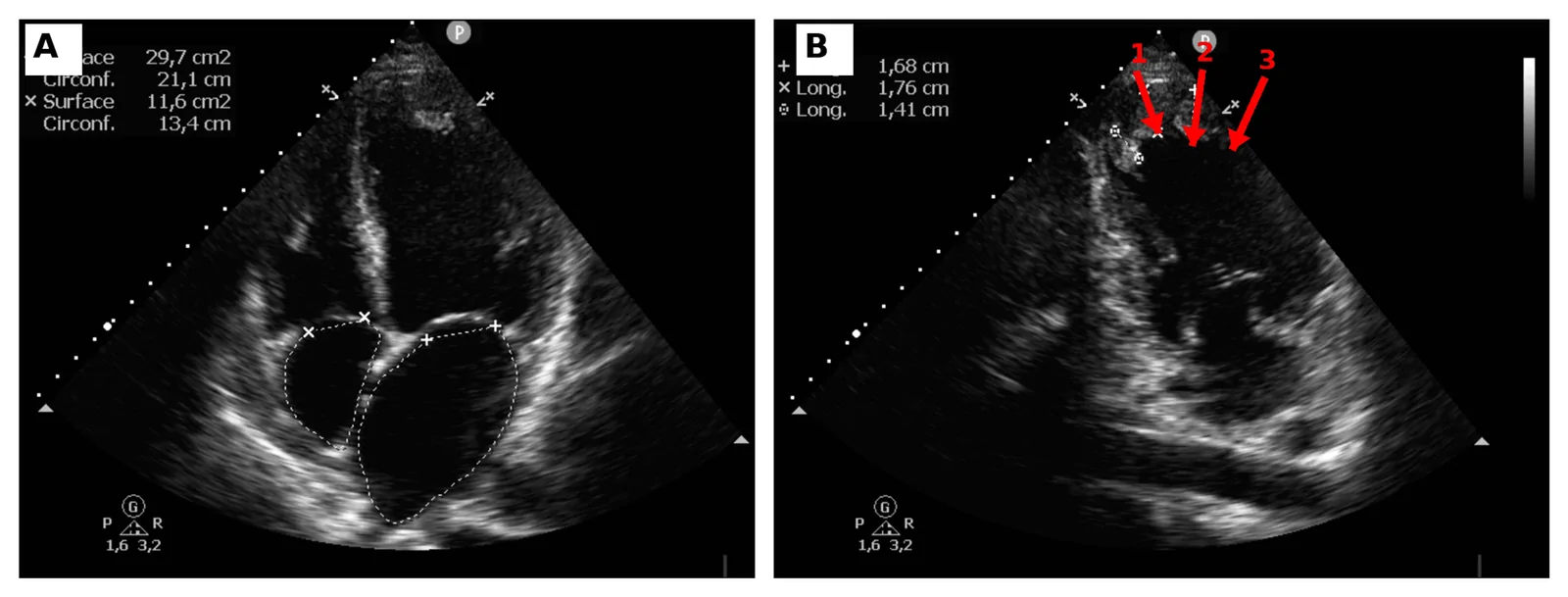
Transthoracic echocardiographic findings. (A) Apical four-chamber view demonstrating enlargement of the left-sided cardiac chambers. (B) Apical view showing three measured formations attached to the left ventricular apex, corresponding to the reported apical thrombi.

**Figure 2:**
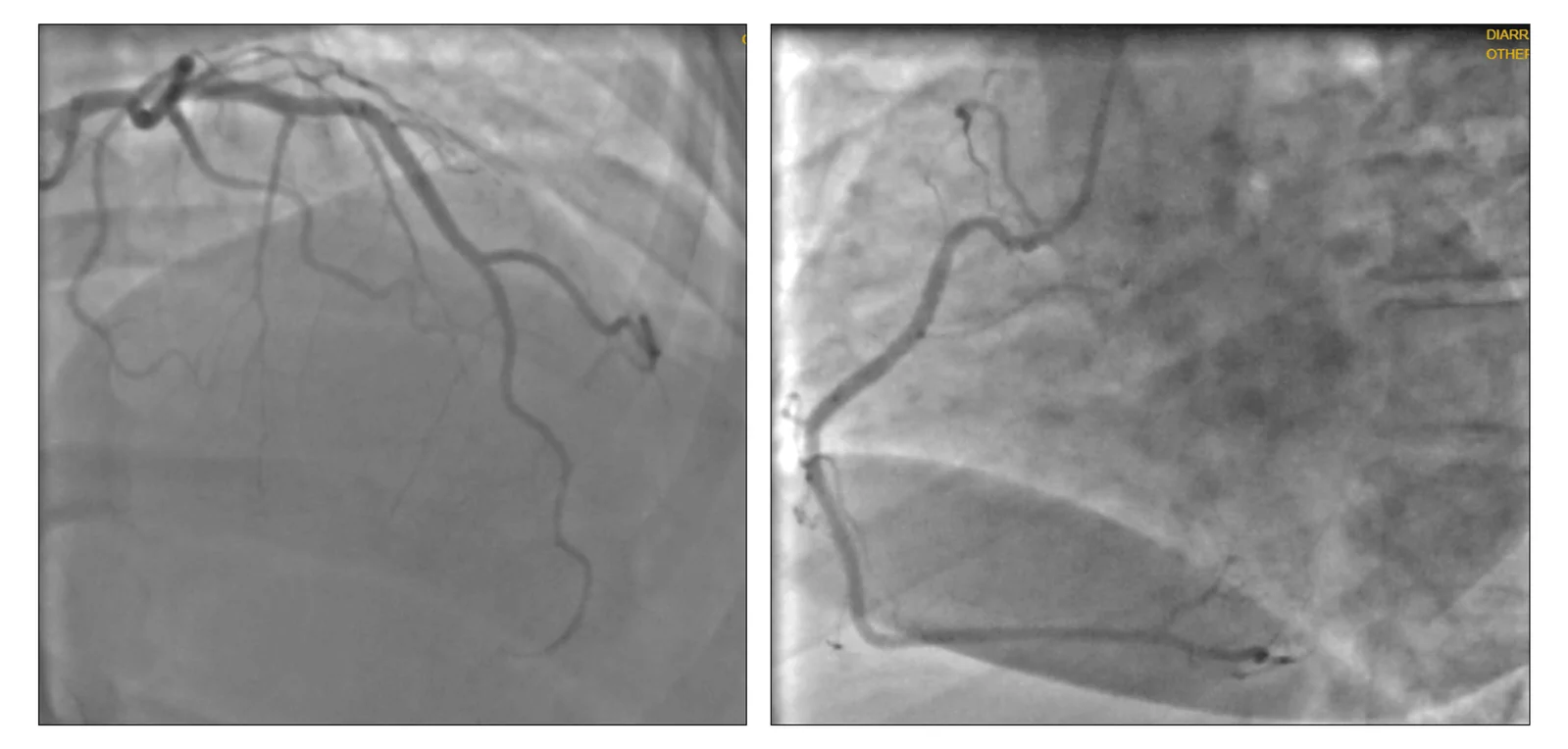
Coronary angiography. Left coronary angiography shows patent left coronary arteries without significant obstructive stenosis. Right coronary angiography shows a patent right coronary artery without significant obstructive stenosis.

On Day 7, CMR demonstrated predominantly apical left ventricular endomyocardial involvement, with marked subendocardial late gadolinium enhancement predominantly involving the apical segments and particularly pronounced at the apex. These findings were associated with apical obliteration and intracavitary thrombi that showed no gadolinium enhancement, allowing differentiation of the thrombi from the enhancing fibrotic endomyocardium. The overall CMR pattern strongly supported the diagnosis of endomyocardial fibrosis (Figure 3). Quantitative CMR parameters, T1 mapping, and extracellular volume measurements were not available for retrospective analysis.

**Figure 3:**
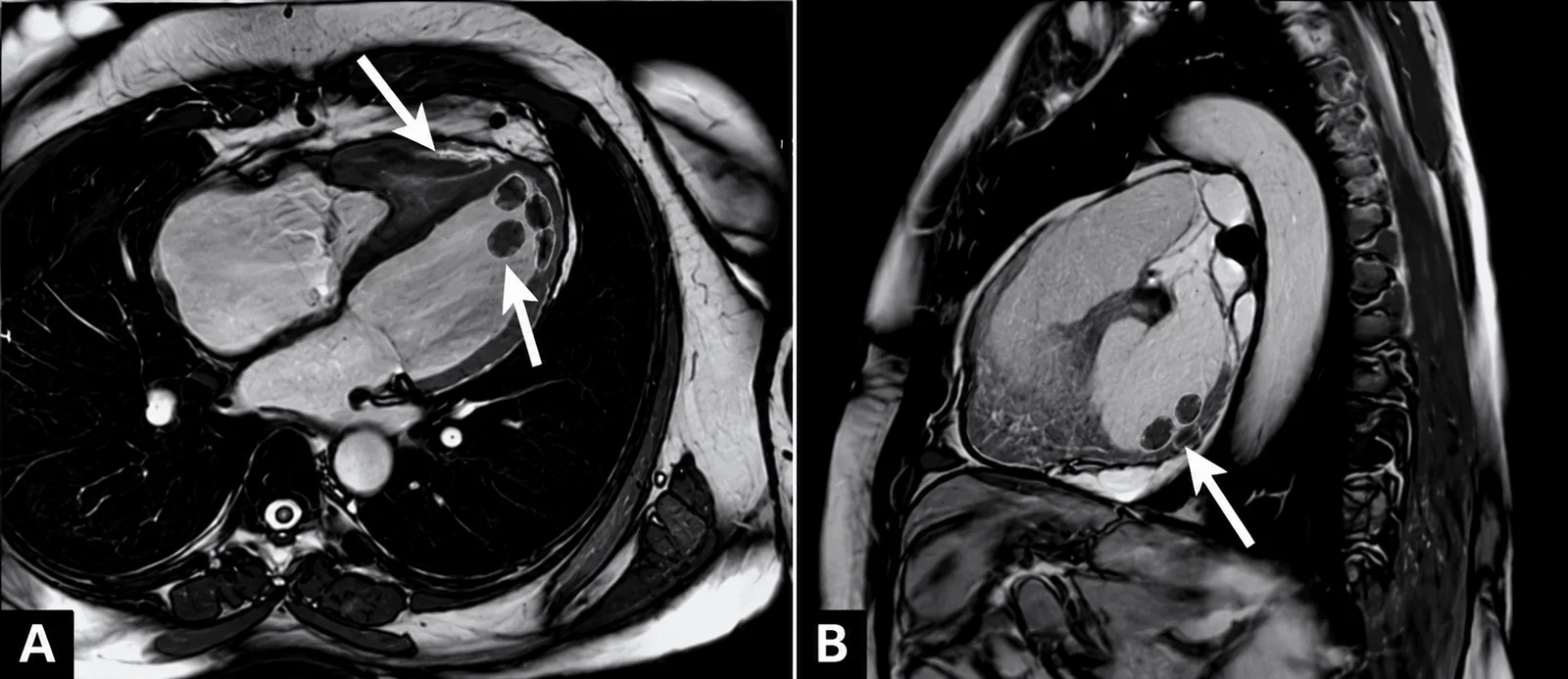
CMR imaging. Available CMR images demonstrate apical left ventricular involvement and intracavitary apical thrombi. The complete CMR examination demonstrates predominantly apical endomyocardial involvement with subendocardial LGE and apical obliteration; the corresponding LGE images were not available for retrospective inclusion. CMR: cardiac magnetic resonance; LGE: late gadolinium enhancement

Given the patient’s relatively young age, atypical epidemiological setting, and family history of cardiomyopathy, he was referred to the Internal Medicine Department for further etiological evaluation and genetic assessment. The results of these investigations were not available to the authors for retrospective analysis.

At three-month follow-up, repeat echocardiography showed persistence of the left ventricular apical thrombi, and left ventricular systolic function had not fully recovered. Detailed quantitative echocardiographic measurements and the original follow-up images were not available for retrospective analysis.

The day-by-day summary of the patient's clinical timeline is presented in Table 2. 

**Table 2:** Clinical timeline ECG: electrocardiography; LVEF: left ventricular ejection fraction; EMF: endomyocardial fibrosis

Day	Clinical event/investigation	Main finding/management
Day 1	Hospital admission	Progressive dyspnea and congestive heart failure; initial clinical, ECG, and laboratory assessment.
Day 3	Transthoracic echocardiography	LVEF 27%, left-sided chamber enlargement, and three apical thrombi. Vitamin K antagonist anticoagulation initiated.
Day 5	Coronary angiography	No significant obstructive coronary artery disease.
Day 7	Cardiac magnetic resonance imaging	Findings consistent with EMF; apical thrombi confirmed.
Day 14	Hospital discharge	Clinical improvement; heart failure therapy and anticoagulation continued.

## Discussion

EMF is classically associated with tropical regions, with a particularly high burden reported in sub-Saharan Africa [1,2,5,8]. In North Africa, the disease is much less frequently reported, although cases and series from Tunisia and Egypt confirm that EMF also occurs in the region [3,4]. Our case is unusual because it concerns a Maghrebi adult living in an urban area, without recent travel to a tropical endemic region and without peripheral eosinophilia.

The etiology and pathophysiology of EMF remain incompletely understood. Environmental, nutritional, immunological, infectious, and genetic mechanisms have all been proposed [5,6]. In our patient, no peripheral eosinophilia was documented during hospitalization. Because of his young age and family history of dilated cardiomyopathy in his sister, an underlying familial or genetic cardiomyopathy was also considered. The patient was referred to internal medicine for further etiological evaluation and genetic assessment; however, the results of this work-up were not available to us. Therefore, a specific underlying cause could not be established.

The differential diagnosis was particularly important because severe global LV systolic dysfunction and multiple apical thrombi are not specific to EMF. Ischemic cardiomyopathy was considered unlikely because coronary angiography showed no significant obstructive coronary artery disease. Other possibilities included myocarditis, eosinophilic heart disease, familial or genetic dilated cardiomyopathy, and inflammatory or infiltrative cardiomyopathies. The available investigations did not establish these alternative diagnoses, although their complete exclusion was limited by the absence of the subsequent etiological and genetic work-up.

Although EMF is classically described in tropical regions, it should also be considered in restrictive cardiomyopathy outside endemic areas, particularly when imaging shows apical involvement or intracavitary thrombi [10]. Echocardiography remains the first-line examination, whereas CMR provides better tissue characterization when echocardiographic findings are insufficient or atypical. In our patient, CMR showed predominantly apical endomyocardial involvement, with marked subendocardial late gadolinium enhancement in the apical segments, associated with apical obliteration. The intracavitary apical thrombi did not enhance, helping to distinguish them from the fibrotic endocardium. These findings supported the diagnosis of EMF in the appropriate clinical context [9,10].

Similar presentations have been reported outside endemic regions. Théry et al. described a 44-year-old French man who had never travelled outside France and in whom heart failure led to the diagnosis of idiopathic EMF by CMR, without eosinophilia or an identified cause [11]. Wagner et al. also reported a 40-year-old Austrian woman without tropical exposure, whose CMR demonstrated subendocardial fibrosis associated with an apical thrombus [12]. Together with our case, these observations show that the absence of tropical exposure or eosinophilia should not exclude EMF when the morphological phenotype and imaging findings are suggestive.

Management depends on the clinical stage and associated complications. Our patient received standard heart failure therapy together with vitamin K antagonist anticoagulation because of the apical thrombi. At three-month follow-up, the apical thrombi persisted and left ventricular systolic function had not completely recovered, supporting continued anticoagulation and heart failure therapy. Surgery may be considered in selected advanced cases, particularly when severe endocardial fibrosis or valve involvement results in major hemodynamic compromise [6,7].

This case has important limitations. Histological confirmation was not available, complete results of the etiological and genetic investigations could not be retrieved, and detailed longer-term follow-up data were unavailable. Consequently, the diagnosis should be regarded as strongly supported by the clinical and multimodality imaging findings rather than histologically confirmed.

## Conclusions

This case illustrates that EMF can occur in a Maghrebi adult without recent tropical exposure and should not be excluded solely on geographic grounds. Echocardiography identified severe left ventricular dysfunction and multiple apical thrombi, while CMR demonstrated apical endomyocardial involvement and subendocardial late gadolinium enhancement, supporting the diagnosis of EMF in the appropriate clinical context. Recognition of intracardiac thrombi directly influenced management through anticoagulation. Although histological confirmation and complete etiological and genetic investigations were unavailable, multimodality imaging combined with careful assessment of alternative diagnoses can support the diagnosis and guide the management of atypical EMF presentations.
